# Prioritization and sequential exclusion of articles in systematic reviews

**DOI:** 10.1002/cl2.1229

**Published:** 2022-03-27

**Authors:** K. M. Saif‐Ur‐Rahman, Md. Hasan, Shahed Hossain, Iqbal Anwar, Yoshihisa Hirakawa, Hiroshi Yatsuya

**Affiliations:** ^1^ Department of Public Health and Health Systems, Graduate School of Medicine Nagoya University Nagoya Japan; ^2^ Health Systems and Population Studies Division icddr,b Dhaka Bangladesh; ^3^ Department of Public Health and Informatics Bangabandhu Sheikh Mujib Medical University (BSMMU) Dhaka Bangladesh; ^4^ James P. Grant School of Public Health BRAC University Dhaka Bangladesh; ^5^ Department of Public Health Fujita Health University School of Medicine Toyoake Aichi Japan

## Abstract

It is difficult to match the causes of exclusion among two independent review authors after screening the title and abstract or full texts in systematic reviews. We have proposed the prioritization and sequential exclusion approach to reduce the subjectivity in reporting reasons for exclusion. This approach might reduce the burden of mismatched numbers while describing the cause of exclusion.

## 1 METHODS DISCUSSION PAPER

The increase in the number of scientific articles being published in scientific journals is exponential (Bastian et al., 2010). Systematic reviews are the precise collation of all available trials or studies to answer a specific question to provide a systematic summary to the clinicians, policymakers, and researchers that helps them to practice evidence‐based decision making and research development (Cochrane Collaboration, 2008). Systematic reviews have faced different obstacles such as reducing bias in study selection, quality assessment, and grading of evidence, which have been overcome by methodological standardization. The transition of reporting standard of a systematic review with the PRISMA (Preferred Reporting Items for Systematic Review and Meta‐analysis) (Moher et al., 2015; Page et al., 2021) statement and its subsequent extensions for the different forms of systematic reviews (Tricco et al., 2018) has been observed over time. Apart from PRISMA, there are other reporting standards such as ROSES (Reporting standards for Systematic Evidence Syntheses in environmental research) which has been developed by review experts (Haddaway et al., 2018). Like the other components of systematic reviews, experts have tested the different approaches for the screening of the title and abstracts as well (Carter, 2018; Ng et al., 2014). We have noticed that the sequence for excluding articles has not been described anywhere. In systematic reviews, authors usually screen the articles for exclusion in two phases. A summary of excluded articles or detailed listing is mandatory for reporting or publishing reviews. Often the excluded article gets two or more reasons in favor of exclusion. As the screening is done by two independent persons, there must be subjectivity in deciding the cause of exclusion for that specific article. Systematic reviews often include a huge number of articles after an initial search. It is questionable how the independent reviewers match the cause of exclusion after screening and how the matched cause of exclusion is reported in the PRISMA flow diagram. Hence the proposed prioritized sequential exclusion approach can be of use to reduce subjectivity in reporting the cause of exclusion.

Considering the issue, we decided to prioritize the cause of exclusion with sequencing. In Cochrane's systematic review methods, it has been mentioned to exclude articles in order of importance. However, no detailed description has been provided. We named the technique “Prioritization and sequential exclusion” (Saif‐Ur‐Rahman et al., 2021). For example, let's consider that the exclusion criteria are geographic region, study design, relevance to review question, type of articles, and co‐morbidity of participants. Authors will prioritize the exclusion criteria and arrange them sequentially as follows: 1. relevance to review question, 2. geographic region, 3. study design, 4. article type, and 5. co‐morbidity of participants. Each reviewer will exclude the screened articles as per the prioritization. One single article can have two or more criteria for exclusion, but reviewers will mention the cause of exclusion following the sequence as an irrelevance to the review question (if applicable). If the article is not excluded for exclusion criteria 1, then they will look for criteria 2 and so on. Using this pre‐specified prioritization will reduce the dispute in notifying the reason for exclusion. There will be very minimal irrelevance which can be cross‐checked easily. We propose that this exclusion sequence should be described in the protocol. To increase the methodological robustness, review authors might consider an inter‐rater reliability assessment based on the matching number of reasons for exclusion in addition to the overall inter‐rater reliability.

While conducting systematic reviews, we face difficulty in matching the number of reasons for exclusion. For example, the first reviewer may exclude one article for the geographic region while the second reviewer might exclude the same article for study design. At the end of the screening included articles are cross‐matched and the dispute is resolved by expert opinion from the third reviewer. But to summarize the cause of exclusion we experience hardship. Often there is a huge difference in numbers between the reasons for exclusion. For the reporting of excluded items, it takes a laborious process to match the numbers under each theme of exclusion.

This prioritization and sequential exclusion approach might be found effective in reducing the differences of reasons for exclusion. This may reduce the extra workload for matching the causes of exclusion resulting from subjective exclusion by independent reviewers. This approach has been tested only in a couple of reviews. More methodological endeavors such as an experimental approach may require validating this attempt. A validated prioritized sequential exclusion is expected to reduce subjectivity and would contribute to improving the reliability of the systematic review reported.

There are many methodological works on developing different strategies for a systematic review. Still, there are spaces to refine the approaches. We identified difficulty in matching the number of causes of exclusion after screening the title and abstract or full text. To reduce subjectivity in reporting reasons for exclusion, we applied the prioritization and sequential exclusion approach. This approach may reduce the burden of mismatched numbers in reporting of cause of exclusion. The exponential increase in the quantity of knowledge would increase the necessity of systematic reviews. Discussion among this scientific field is needed to validate or modify this approach.

## AUTHOR CONTRIBUTIONS

*Conceptualized the work, wrote the draft and finalized the manuscript*: K. M. Saif‐Ur‐Rahman. *Conceptualized the work and revised the manuscript*: Md. Hasan. *Critically revised the manuscript*: Shahed Hossain, Iqbal Anwar, Yoshihisa Hirakawa, and Hiroshi Yatsuya. Corresponding author is the guarantor of the work. All the authors read and approved the final manuscript.

## CONFLICTS OF INTEREST

The authors declare no conflicts of interest.
